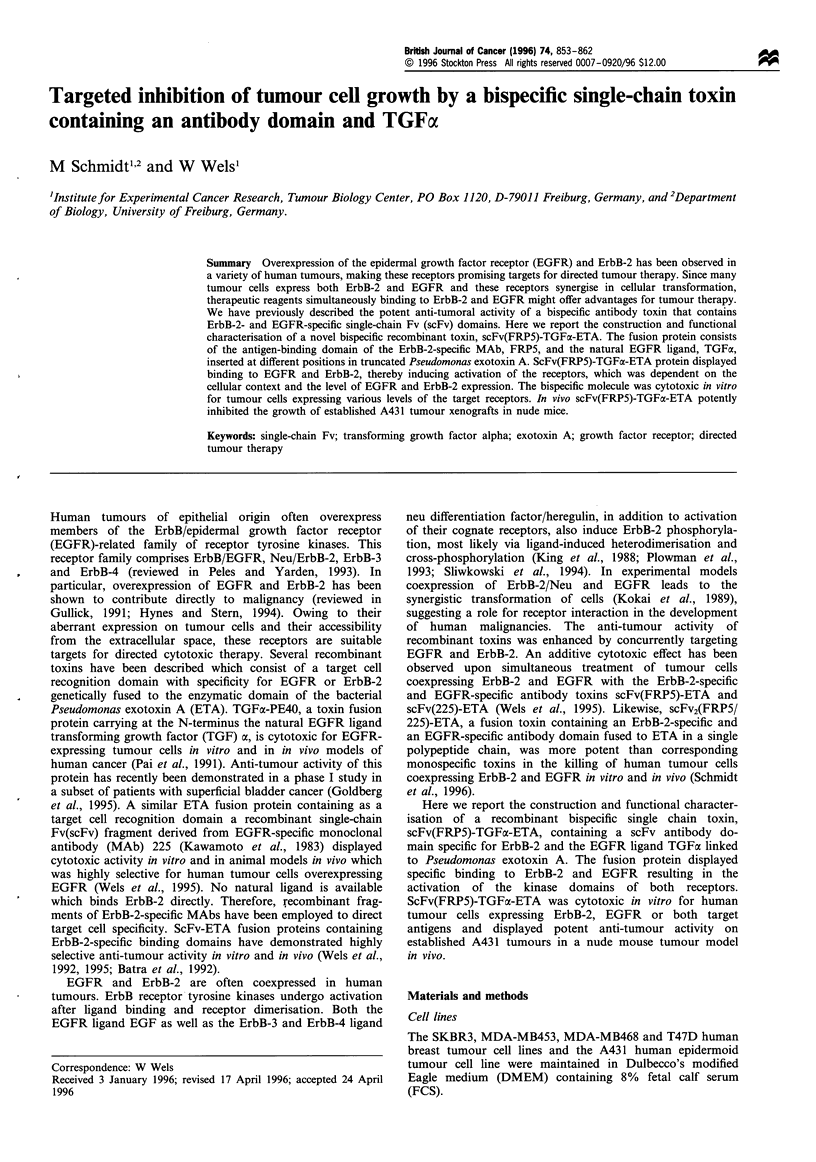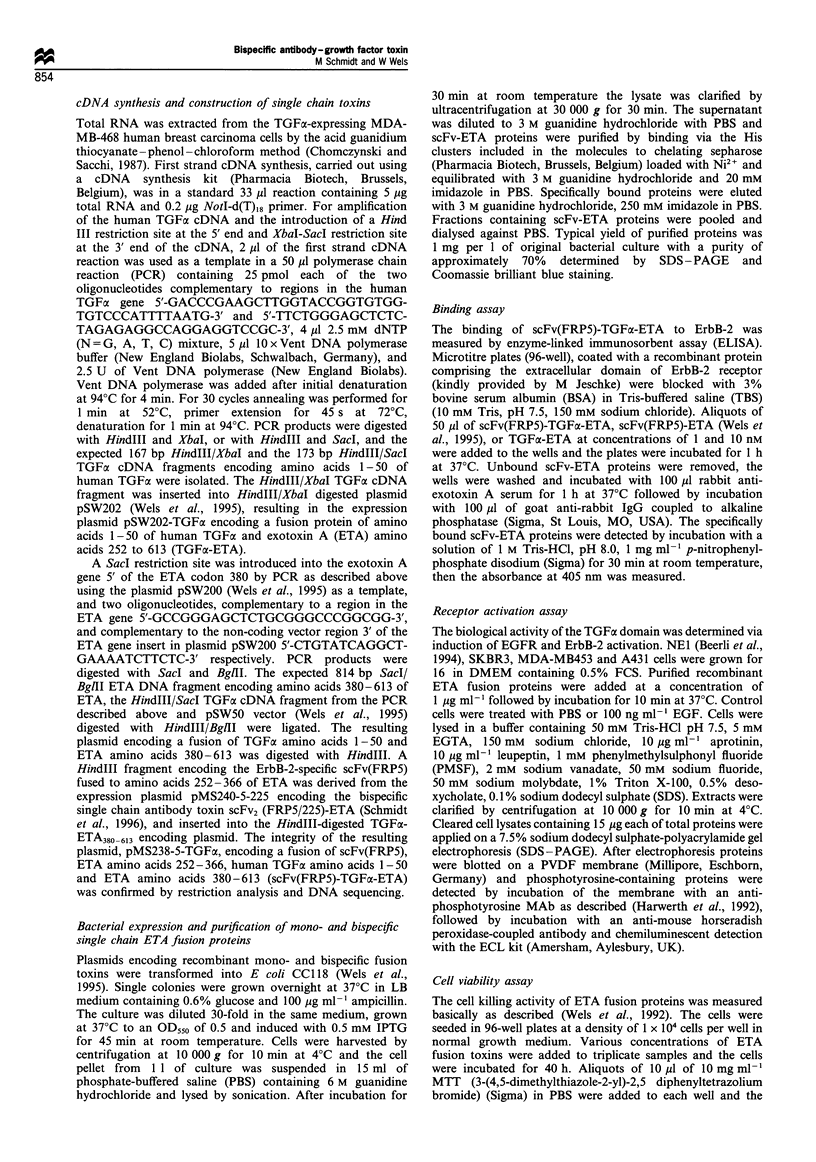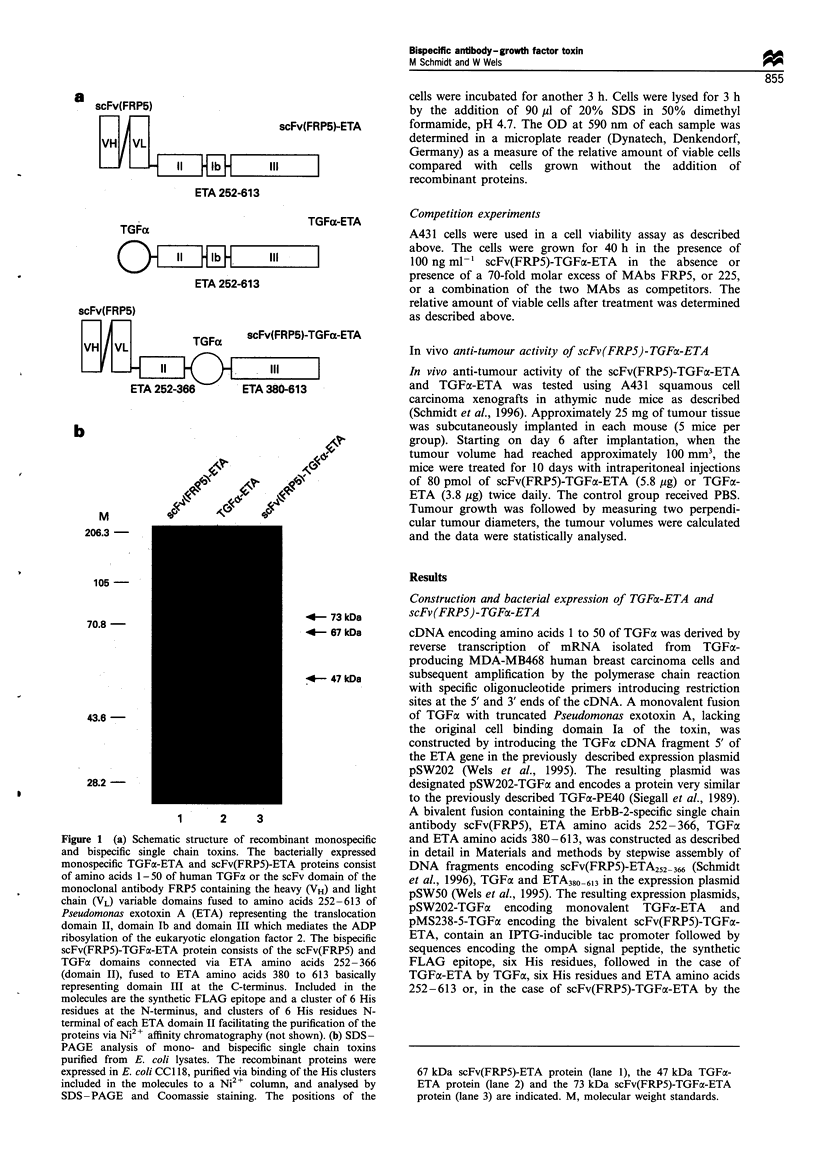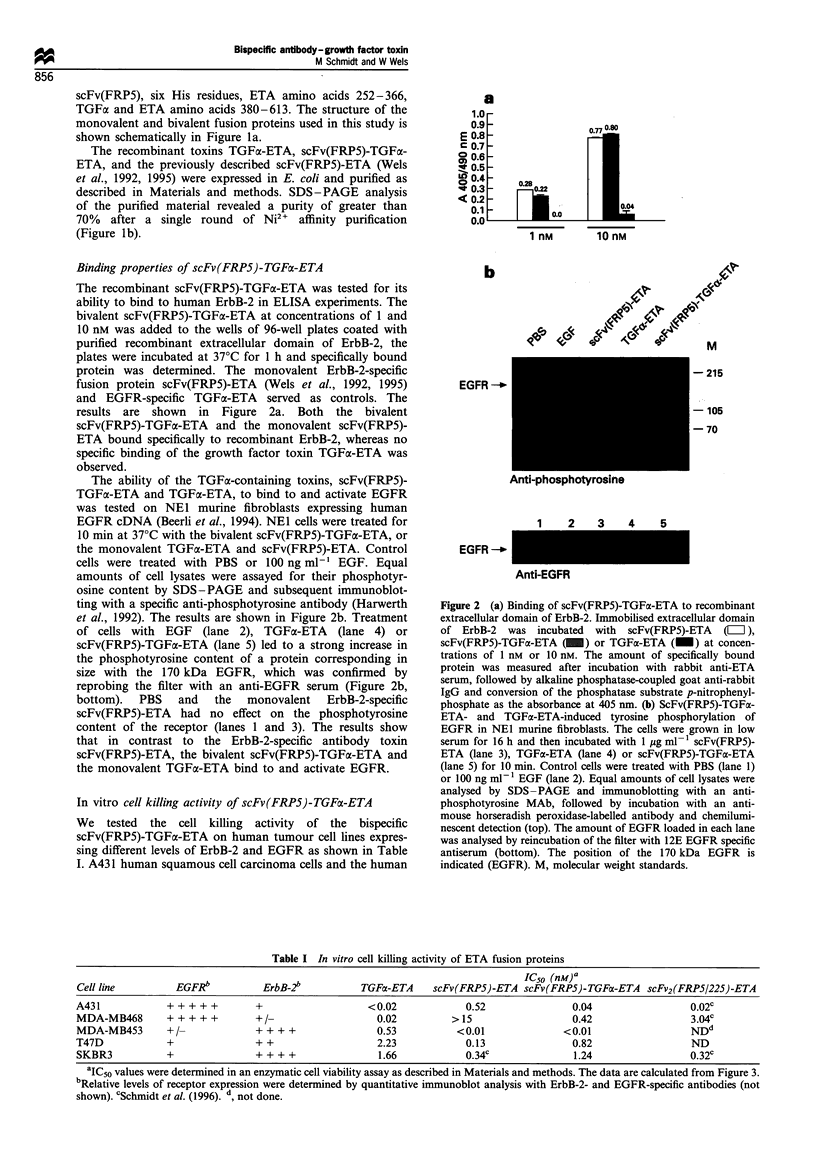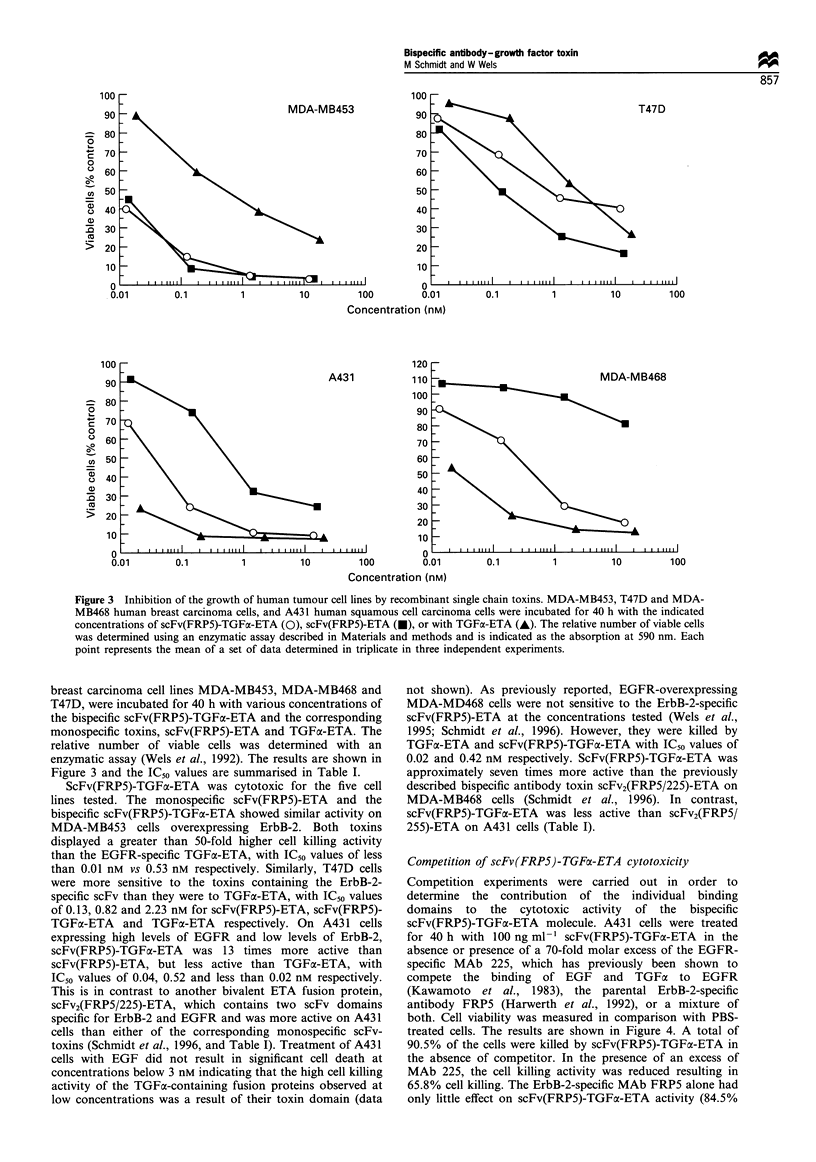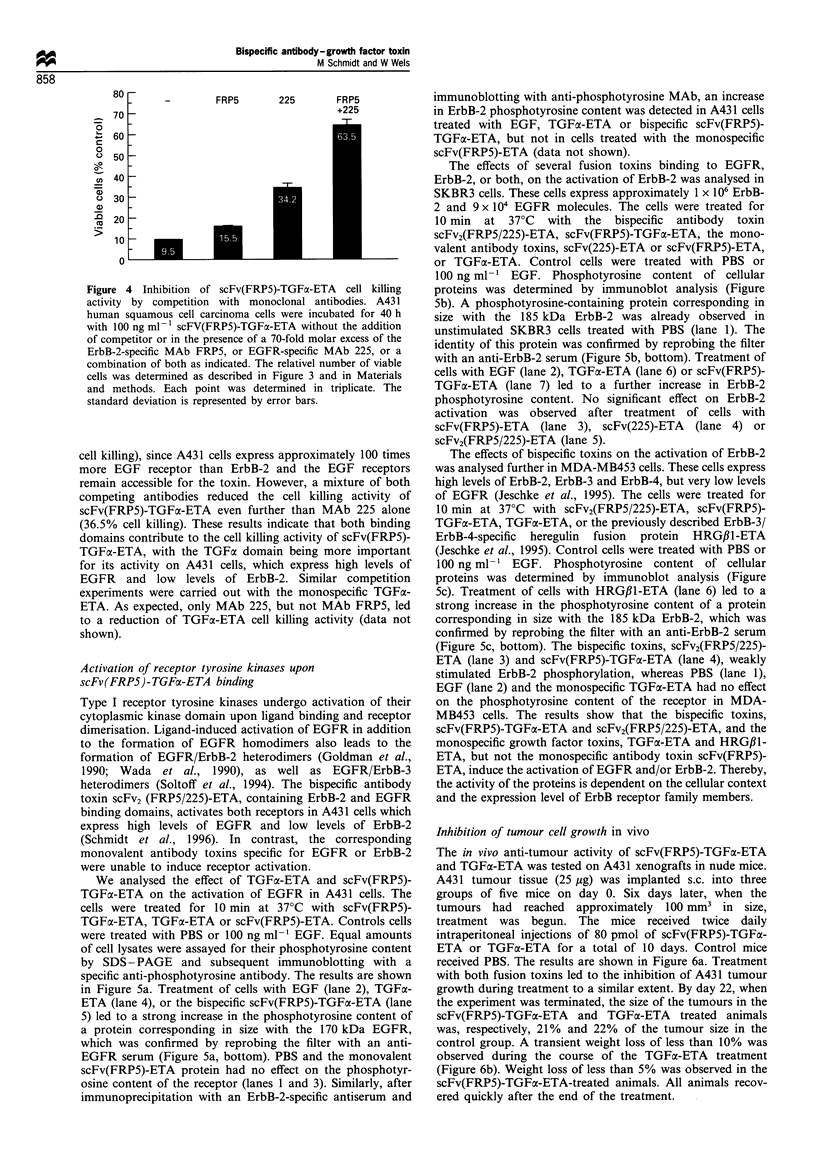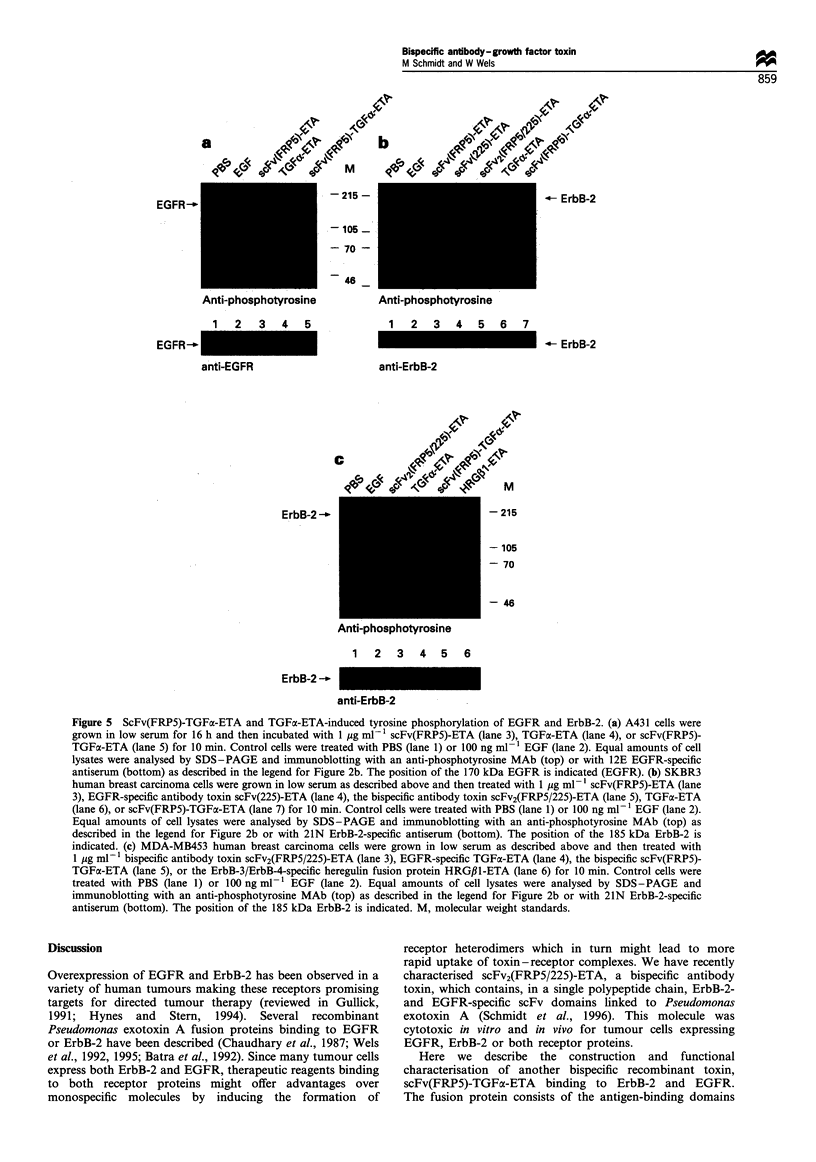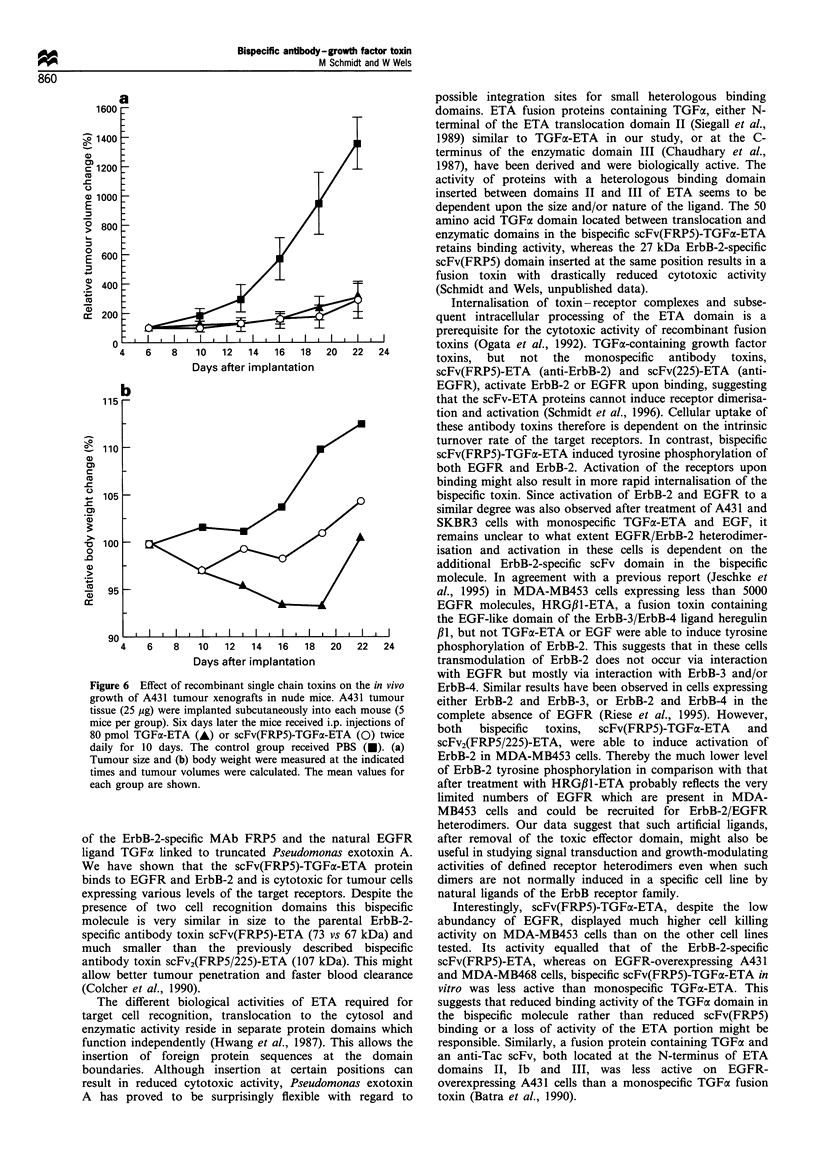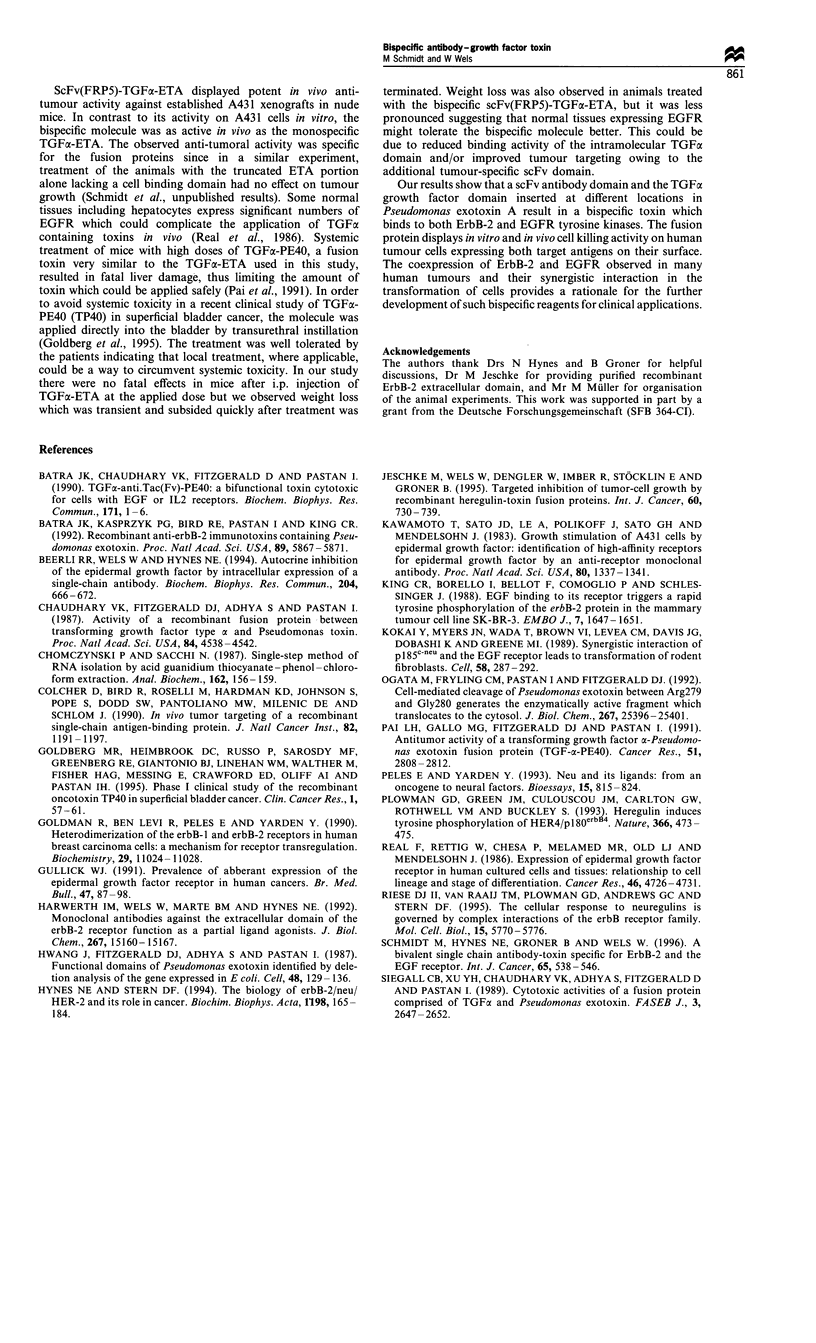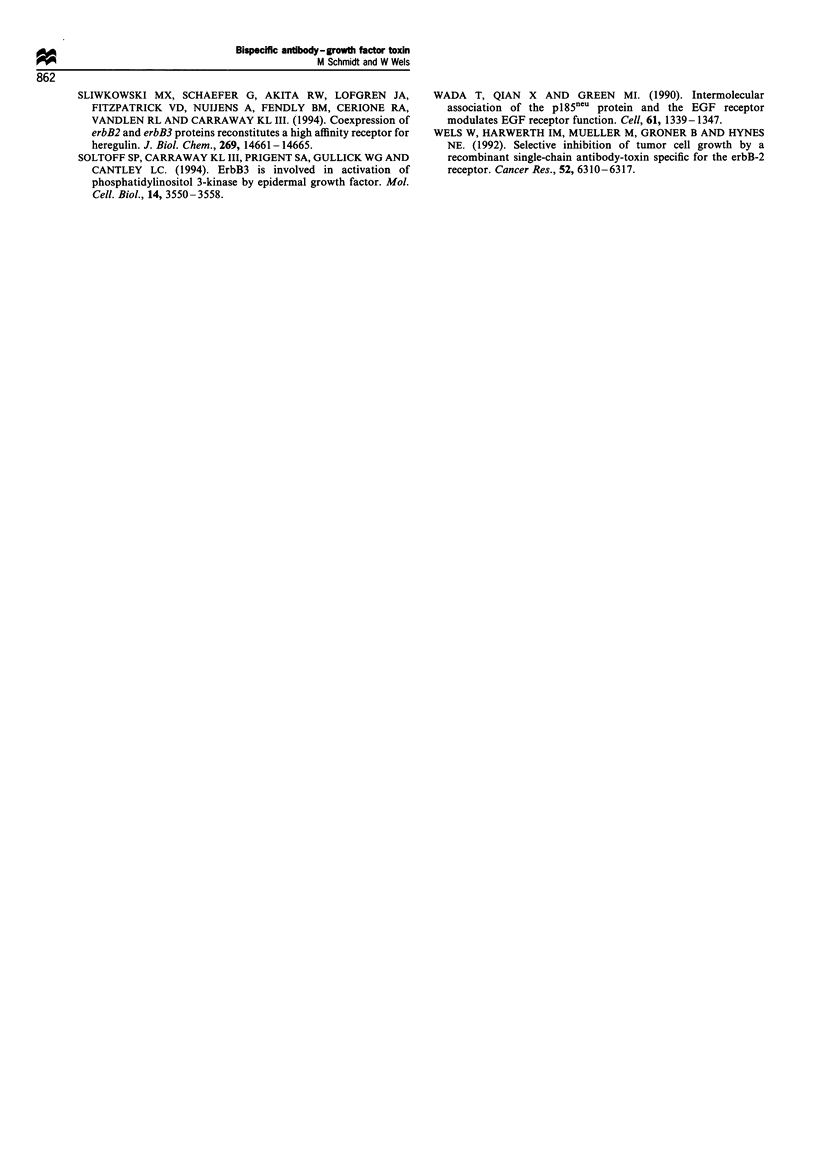# Targeted inhibition of tumour cell growth by a bispecific single-chain toxin containing an antibody domain and TGF alpha.

**DOI:** 10.1038/bjc.1996.448

**Published:** 1996-09

**Authors:** M. Schmidt, W. Wels

**Affiliations:** Institute for Experimental Cancer Research, Freiburg, Germany.

## Abstract

**Images:**


British Journal of Cancer (1996) 74, 853–862
© 1996 Stockton Press All rights reserved 0007-0920/96 $12.00

### Targeted inhibition of tumour cell growth by a bispecific single-chain toxin containing an antibody domain and TGFα

M Schmidt[1,2] and W Wels[1]

[1]*Institute for Experimental Cancer Research, Tumour Biology Center, PO Box 1120, D-79011 Freiburg, Germany, and* [2]*Department of Biology, University of Freiburg, Germany.*

**Summary** Overexpression of the epidermal growth factor receptor (EGFR) and ErbB-2 has been observed in a variety of human tumours, making these receptors promising targets for directed tumour therapy. Since many tumour cells express both ErbB-2 and EGFR and these receptors synergise in cellular transformation, therapeutic reagents simultaneously binding to ErbB-2 and EGFR might offer advantages for tumour therapy. We have previously described the potent anti-tumoral activity of a bispecific antibody toxin that contains ErbB-2- and EGFR-specific single-chain Fv (scFv) domains. Here we report the construction and functional characterisation of a novel bispecific recombinant toxin, scFv(FRP5)-TGFα-ETA. The fusion protein consists of the antigen-binding domain of the ErbB-2-specific MAb, FRP5, and the natural EGFR ligand, TGFα, inserted at different positions in truncated *Pseudomonas* exotoxin A. ScFv(FRP5)-TGFα-ETA protein displayed binding to EGFR and ErbB-2, thereby inducing activation of the receptors, which was dependent on the cellular context and the level of EGFR and ErbB-2 expression. The bispecific molecule was cytotoxic *in vitro* for tumour cells expressing various levels of the target receptors. *In vivo* scFv(FRP5)-TGFα-ETA potently inhibited the growth of established A431 tumour xenografts in nude mice.

**Keywords:** single-chain Fv; transforming growth factor alpha; exotoxin A; growth factor receptor; directed tumour therapy

Human tumours of epithelial origin often overexpress members of the ErbB/epidermal growth factor receptor (EGFR)-related family of receptor tyrosine kinases. This receptor family comprises ErbB/EGFR, Neu/ErbB-2, ErbB-3 and ErbB-4 (reviewed in Peles and Yarden, 1993). In particular, overexpression of EGFR and ErbB-2 has been shown to contribute directly to malignancy (reviewed in Gullick, 1991; Hynes and Stern, 1994). Owing to their aberrant expression on tumour cells and their accessibility from the extracellular space, these receptors are suitable targets for directed cytotoxic therapy. Several recombinant toxins have been described which consist of a target cell recognition domain with specificity for EGFR or ErbB-2 genetically fused to the enzymatic domain of the bacterial *Pseudomonas* exotoxin A (ETA). TGFα-PE40, a toxin fusion protein carrying at the N-terminus the natural EGFR ligand transforming growth factor (TGF) α, is cytotoxic for EGFR-expressing tumour cells *in vitro* and in *in vivo* models of human cancer (Pai *et al.*, 1991). Anti-tumour activity of this protein has recently been demonstrated in a phase I study in a subset of patients with superficial bladder cancer (Goldberg *et al.*, 1995). A similar ETA fusion protein containing as a target cell recognition domain a recombinant single-chain Fv(scFv) fragment derived from EGFR-specific monoclonal antibody (MAb) 225 (Kawamoto *et al.*, 1983) displayed cytotoxic activity *in vitro* and in animal models *in vivo* which was highly selective for human tumour cells overexpressing EGFR (Wels *et al.*, 1995). No natural ligand is available which binds ErbB-2 directly. Therefore, recombinant fragments of ErbB-2-specific MAbs have been employed to direct target cell specificity. ScFv-ETA fusion proteins containing ErbB-2-specific binding domains have demonstrated highly selective anti-tumour activity *in vitro* and *in vivo* (Wels *et al.*, 1992, 1995; Batra *et al.*, 1992).

EGFR and ErbB-2 are often coexpressed in human tumours. ErbB receptor tyrosine kinases undergo activation after ligand binding and receptor dimerisation. Both the EGFR ligand EGF as well as the ErbB-3 and ErbB-4 ligand neu differentiation factor/heregulin, in addition to activation of their cognate receptors, also induce ErbB-2 phosphorylation, most likely via ligand-induced heterodimerisation and cross-phosphorylation (King *et al.*, 1988; Plowman *et al.*, 1993; Sliwkowski *et al.*, 1994). In experimental models coexpression of ErbB-2/Neu and EGFR leads to the synergistic transformation of cells (Kokai *et al.*, 1989), suggesting a role for receptor interaction in the development of human malignancies. The anti-tumour activity of recombinant toxins was enhanced by concurrently targeting EGFR and ErbB-2. An additive cytotoxic effect has been observed upon simultaneous treatment of tumour cells coexpressing ErbB-2 and EGFR with the ErbB-2-specific and EGFR-specific antibody toxins scFv(FRP5)-ETA and scFv(225)-ETA (Wels *et al.*, 1995). Likewise, $scFv_2$(FRP5/225)-ETA, a fusion toxin containing an ErbB-2-specific and an EGFR-specific antibody domain fused to ETA in a single polypeptide chain, was more potent than corresponding monospecific toxins in the killing of human tumour cells coexpressing ErbB-2 and EGFR *in vitro* and *in vivo* (Schmidt *et al.*, 1996).

Here we report the construction and functional characterisation of a recombinant bispecific single chain toxin, scFv(FRP5)-TGFα-ETA, containing a scFv antibody domain specific for ErbB-2 and the EGFR ligand TGFα linked to *Pseudomonas* exotoxin A. The fusion protein displayed specific binding to ErbB-2 and EGFR resulting in the activation of the kinase domains of both receptors. ScFv(FRP5)-TGFα-ETA was cytotoxic *in vitro* for human tumour cells expressing ErbB-2, EGFR or both target antigens and displayed potent anti-tumour activity on established A431 tumours in a nude mouse tumour model *in vivo*.

### Materials and methods

### Cell lines

The SKBR3, MDA-MB453, MDA-MB468 and T47D human breast tumour cell lines and the A431 human epidermoid tumour cell line were maintained in Dulbecco's modified Eagle medium (DMEM) containing 8% fetal calf serum (FCS).

Correspondence: W Wels
Received 3 January 1996; revised 17 April 1996; accepted 24 April 1996

*cDNA synthesis and construction of single chain toxins*

Total RNA was extracted from the TGFα-expressing MDA-MB-468 human breast carcinoma cells by the acid guanidium thiocyanate-phenol-chloroform method (Chomczynski and Sacchi, 1987). First strand cDNA synthesis, carried out using a cDNA synthesis kit (Pharmacia Biotech, Brussels, Belgium), was in a standard 33 μl reaction containing 5 μg total RNA and 0.2 μg *Not*I-d$(T)_{18}$ primer. For amplification of the human TGFα cDNA and the introduction of a *Hind* III restriction site at the 5′ end and *Xba*I-*Sac*I restriction site at the 3′ end of the cDNA, 2 μl of the first strand cDNA reaction was used as a template in a 50 μl polymerase chain reaction (PCR) containing 25 pmol each of the two oligonucleotides complementary to regions in the human TGFα gene 5′-GACCCGAAGCTTGGTACCGGTGTGG-TGTCCCATTTTAATG-3′ and 5′-TTCTGGGAGCTCTC-TAGAGAGGCCAGGAGGTCCGC-3′, 4 μl 2.5 mM dNTP (N=G, A, T, C) mixture, 5 μl 10×Vent DNA polymerase buffer (New England Biolabs, Schwalbach, Germany), and 2.5 U of Vent DNA polymerase (New England Biolabs). Vent DNA polymerase was added after initial denaturation at 94°C for 4 min. For 30 cycles annealing was performed for 1 min at 52°C, primer extension for 45 s at 72°C, denaturation for 1 min at 94°C. PCR products were digested with *Hind*III and *Xba*I, or with *Hind*III and *Sac*I, and the expected 167 bp *Hind*III/*Xba*I and the 173 bp *Hind*III/*Sac*I TGFα cDNA fragments encoding amino acids 1-50 of human TGFα were isolated. The *Hind*III/*Xba*I TGFα cDNA fragment was inserted into *Hind*III/*Xba*I digested plasmid pSW202 (Wels *et al.*, 1995), resulting in the expression plasmid pSW202-TGFα encoding a fusion protein of amino acids 1-50 of human TGFα and exotoxin A (ETA) amino acids 252 to 613 (TGFα-ETA).

A *Sac*I restriction site was introduced into the exotoxin A gene 5′ of the ETA codon 380 by PCR as described above using the plasmid pSW200 (Wels *et al.*, 1995) as a template, and two oligonucleotides, complementary to a region in the ETA gene 5′-GCCGGGAGCTCTGCGGGCCCGGCGG-3′, and complementary to the non-coding vector region 3′ of the ETA gene insert in plasmid pSW200 5′-CTGTATCAGGCT-GAAAATCTTCTC-3′ respectively. PCR products were digested with *Sac*I and *Bgl*II. The expected 814 bp *Sac*I/*Bgl*II ETA DNA fragment encoding amino acids 380-613 of ETA, the *Hind*III/*Sac*I TGFα cDNA fragment from the PCR described above and pSW50 vector (Wels *et al.*, 1995) digested with *Hind*III/*Bgl*II were ligated. The resulting plasmid encoding a fusion of TGFα amino acids 1-50 and ETA amino acids 380-613 was digested with *Hind*III. A *Hind*III fragment encoding the ErbB-2-specific scFv(FRP5) fused to amino acids 252-366 of ETA was derived from the expression plasmid pMS240-5-225 encoding the bispecific single chain antibody toxin $scFv_2$ (FRP5/225)-ETA (Schmidt *et al.*, 1996), and inserted into the *Hind*III-digested TGFα-$ETA_{380-613}$ encoding plasmid. The integrity of the resulting plasmid, pMS238-5-TGFα, encoding a fusion of scFv(FRP5), ETA amino acids 252-366, human TGFα amino acids 1-50 and ETA amino acids 380-613 (scFv(FRP5)-TGFα-ETA) was confirmed by restriction analysis and DNA sequencing.

*Bacterial expression and purification of mono- and bispecific single chain ETA fusion proteins*

Plasmids encoding recombinant mono- and bispecific fusion toxins were transformed into *E coli* CC118 (Wels *et al.*, 1995). Single colonies were grown overnight at 37°C in LB medium containing 0.6% glucose and 100 μg $ml^{-1}$ ampicillin. The culture was diluted 30-fold in the same medium, grown at 37°C to an $OD_{550}$ of 0.5 and induced with 0.5 mM IPTG for 45 min at room temperature. Cells were harvested by centrifugation at 10 000 *g* for 10 min at 4°C and the cell pellet from 1 l of culture was suspended in 15 ml of phosphate-buffered saline (PBS) containing 6 M guanidine hydrochloride and lysed by sonication. After incubation for 30 min at room temperature the lysate was clarified by ultracentrifugation at 30 000 *g* for 30 min. The supernatant was diluted to 3 M guanidine hydrochloride with PBS and scFv-ETA proteins were purified by binding via the His clusters included in the molecules to chelating sepharose (Pharmacia Biotech, Brussels, Belgium) loaded with $Ni^{2+}$ and equilibrated with 3 M guanidine hydrochloride and 20 mM imidazole in PBS. Specifically bound proteins were eluted with 3 M guanidine hydrochloride, 250 mM imidazole in PBS. Fractions containing scFv-ETA proteins were pooled and dialysed against PBS. Typical yield of purified proteins was 1 mg per l of original bacterial culture with a purity of approximately 70% determined by SDS-PAGE and Coomassie brilliant blue staining.

*Binding assay*

The binding of scFv(FRP5)-TGFα-ETA to ErbB-2 was measured by enzyme-linked immunosorbent assay (ELISA). Microtitre plates (96-well), coated with a recombinant protein comprising the extracellular domain of ErbB-2 receptor (kindly provided by M Jeschke) were blocked with 3% bovine serum albumin (BSA) in Tris-buffered saline (TBS) (10 mM Tris, pH 7.5, 150 mM sodium chloride). Aliquots of 50 μl of scFv(FRP5)-TGFα-ETA, scFv(FRP5)-ETA (Wels *et al.*, 1995), or TGFα-ETA at concentrations of 1 and 10 nM were added to the wells and the plates were incubated for 1 h at 37°C. Unbound scFv-ETA proteins were removed, the wells were washed and incubated with 100 μl rabbit anti-exotoxin A serum for 1 h at 37°C followed by incubation with 100 μl of goat anti-rabbit IgG coupled to alkaline phosphatase (Sigma, St Louis, MO, USA). The specifically bound scFv-ETA proteins were detected by incubation with a solution of 1 M Tris-HCl, pH 8.0, 1 mg $ml^{-1}$ *p*-nitrophenyl-phosphate disodium (Sigma) for 30 min at room temperature, then the absorbance at 405 nm was measured.

*Receptor activation assay*

The biological activity of the TGFα domain was determined via induction of EGFR and ErbB-2 activation. NE1 (Beerli *et al.*, 1994), SKBR3, MDA-MB453 and A431 cells were grown for 16 in DMEM containing 0.5% FCS. Purified recombinant ETA fusion proteins were added at a concentration of 1 μg $ml^{-1}$ followed by incubation for 10 min at 37°C. Control cells were treated with PBS or 100 ng $ml^{-1}$ EGF. Cells were lysed in a buffer containing 50 mM Tris-HCl pH 7.5, 5 mM EGTA, 150 mM sodium chloride, 10 μg $ml^{-1}$ aprotinin, 10 μg $ml^{-1}$ leupeptin, 1 mM phenylmethylsulphonyl fluoride (PMSF), 2 mM sodium vanadate, 50 mM sodium fluoride, 50 mM sodium molybdate, 1% Triton X-100, 0.5% desoxycholate, 0.1% sodium dodecyl sulphate (SDS). Extracts were clarified by centrifugation at 10 000 *g* for 10 min at 4°C. Cleared cell lysates containing 15 μg each of total proteins were applied on a 7.5% sodium dodecyl sulphate-polyacrylamide gel electrophoresis (SDS-PAGE). After electrophoresis proteins were blotted on a PVDF membrane (Millipore, Eschborn, Germany) and phosphotyrosine-containing proteins were detected by incubation of the membrane with an anti-phosphotyrosine MAb as described (Harwerth *et al.*, 1992), followed by incubation with an anti-mouse horseradish peroxidase-coupled antibody and chemiluminescent detection with the ECL kit (Amersham, Aylesbury, UK).

*Cell viability assay*

The cell killing activity of ETA fusion proteins was measured basically as described (Wels *et al.*, 1992). The cells were seeded in 96-well plates at a density of $1 \times 10^4$ cells per well in normal growth medium. Various concentrations of ETA fusion toxins were added to triplicate samples and the cells were incubated for 40 h. Aliquots of 10 μl of 10 mg $ml^{-1}$ MTT (3-(4,5-dimethylthiazole-2-yl)-2,5 diphenyltetrazolium bromide) (Sigma) in PBS were added to each well and the

**Figure 1** (**a**) Schematic structure of recombinant monospecific and bispecific single chain toxins. The bacterially expressed monospecific TGFα-ETA and scFv(FRP5)-ETA proteins consist of amino acids 1–50 of human TGFα or the scFv domain of the monoclonal antibody FRP5 containing the heavy ($V_H$) and light chain ($V_L$) variable domains fused to amino acids 252–613 of *Pseudomonas* exotoxin A (ETA) representing the translocation domain II, domain Ib and domain III which mediates the ADP ribosylation of the eukaryotic elongation factor 2. The bispecific scFv(FRP5)-TGFα-ETA protein consists of the scFv(FRP5) and TGFα domains connected via ETA amino acids 252–366 (domain II), fused to ETA amino acids 380 to 613 basically representing domain III at the C-terminus. Included in the molecules are the synthetic FLAG epitope and a cluster of 6 His residues at the N-terminus, and clusters of 6 His residues N-terminal of each ETA domain II facilitating the purification of the proteins via $Ni^{2+}$ affinity chromatography (not shown). (**b**) SDS–PAGE analysis of mono- and bispecific single chain toxins purified from *E. coli* lysates. The recombinant proteins were expressed in *E. coli* CC118, purified via binding of the His clusters included in the molecules to a $Ni^{2+}$ column, and analysed by SDS–PAGE and Coomassie staining. The positions of the 67 kDa scFv(FRP5)-ETA protein (lane 1), the 47 kDa TGFα-ETA protein (lane 2) and the 73 kDa scFv(FRP5)-TGFα-ETA protein (lane 3) are indicated. M, molecular weight standards.

cells were incubated for another 3 h. Cells were lysed for 3 h by the addition of 90 μl of 20% SDS in 50% dimethyl formamide, pH 4.7. The OD at 590 nm of each sample was determined in a microplate reader (Dynatech, Denkendorf, Germany) as a measure of the relative amount of viable cells compared with cells grown without the addition of recombinant proteins.

### *Competition experiments*

A431 cells were used in a cell viability assay as described above. The cells were grown for 40 h in the presence of 100 ng $ml^{-1}$ scFv(FRP5)-TGFα-ETA in the absence or presence of a 70-fold molar excess of MAbs FRP5, or 225, or a combination of the two MAbs as competitors. The relative amount of viable cells after treatment was determined as described above.

### In vivo *anti-tumour activity of scFv(FRP5)-TGFα-ETA*

*In vivo* anti-tumour activity of the scFv(FRP5)-TGFα-ETA and TGFα-ETA was tested using A431 squamous cell carcinoma xenografts in athymic nude mice as described (Schmidt *et al.*, 1996). Approximately 25 mg of tumour tissue was subcutaneously implanted in each mouse (5 mice per group). Starting on day 6 after implantation, when the tumour volume had reached approximately 100 $mm^3$, the mice were treated for 10 days with intraperitoneal injections of 80 pmol of scFv(FRP5)-TGFα-ETA (5.8 μg) or TGFα-ETA (3.8 μg) twice daily. The control group received PBS. Tumour growth was followed by measuring two perpendicular tumour diameters, the tumour volumes were calculated and the data were statistically analysed.

### Results

### *Construction and bacterial expression of TGFα-ETA and scFv(FRP5)-TGFα-ETA*

cDNA encoding amino acids 1 to 50 of TGFα was derived by reverse transcription of mRNA isolated from TGFα-producing MDA-MB468 human breast carcinoma cells and subsequent amplification by the polymerase chain reaction with specific oligonucleotide primers introducing restriction sites at the 5′ and 3′ ends of the cDNA. A monovalent fusion of TGFα with truncated *Pseudomonas* exotoxin A, lacking the original cell binding domain Ia of the toxin, was constructed by introducing the TGFα cDNA fragment 5′ of the ETA gene in the previously described expression plasmid pSW202 (Wels *et al.*, 1995). The resulting plasmid was designated pSW202-TGFα and encodes a protein very similar to the previously described TGFα-PE40 (Siegall *et al.*, 1989). A bivalent fusion containing the ErbB-2-specific single chain antibody scFv(FRP5), ETA amino acids 252–366, TGFα and ETA amino acids 380–613, was constructed as described in detail in Materials and methods by stepwise assembly of DNA fragments encoding scFv(FRP5)-$ETA_{252-366}$ (Schmidt *et al.*, 1996), TGFα and $ETA_{380-613}$ in the expression plasmid pSW50 (Wels *et al.*, 1995). The resulting expression plasmids, pSW202-TGFα encoding monovalent TGFα-ETA and pMS238-5-TGFα encoding the bivalent scFv(FRP5)-TGFα-ETA, contain an IPTG-inducible tac promoter followed by sequences encoding the ompA signal peptide, the synthetic FLAG epitope, six His residues, followed in the case of TGFα-ETA by TGFα, six His residues and ETA amino acids 252–613 or, in the case of scFv(FRP5)-TGFα-ETA by the scFv(FRP5), six His residues, ETA amino acids 252–366, TGFα and ETA amino acids 380–613. The structure of the monovalent and bivalent fusion proteins used in this study is shown schematically in Figure 1a.

The recombinant toxins TGFα-ETA, scFv(FRP5)-TGFα-ETA, and the previously described scFv(FRP5)-ETA (Wels *et al.*, 1992, 1995) were expressed in *E. coli* and purified as described in Materials and methods. SDS–PAGE analysis of the purified material revealed a purity of greater than 70% after a single round of $Ni^{2+}$ affinity purification (Figure 1b).

### *Binding properties of scFv(FRP5)-TGFα-ETA*

The recombinant scFv(FRP5)-TGFα-ETA was tested for its ability to bind to human ErbB-2 in ELISA experiments. The bivalent scFv(FRP5)-TGFα-ETA at concentrations of 1 and 10 nM was added to the wells of 96-well plates coated with purified recombinant extracellular domain of ErbB-2, the plates were incubated at 37°C for 1 h and specifically bound protein was determined. The monovalent ErbB-2-specific fusion protein scFv(FRP5)-ETA (Wels *et al.*, 1992, 1995) and EGFR-specific TGFα-ETA served as controls. The results are shown in Figure 2a. Both the bivalent scFv(FRP5)-TGFα-ETA and the monovalent scFv(FRP5)-ETA bound specifically to recombinant ErbB-2, whereas no specific binding of the growth factor toxin TGFα-ETA was observed.

The ability of the TGFα-containing toxins, scFv(FRP5)-TGFα-ETA and TGFα-ETA, to bind to and activate EGFR was tested on NE1 murine fibroblasts expressing human EGFR cDNA (Beerli *et al.*, 1994). NE1 cells were treated for 10 min at 37°C with the bivalent scFv(FRP5)-TGFα-ETA, or the monovalent TGFα-ETA and scFv(FRP5)-ETA. Control cells were treated with PBS or 100 ng $ml^{-1}$ EGF. Equal amounts of cell lysates were assayed for their phosphotyrosine content by SDS–PAGE and subsequent immunoblotting with a specific anti-phosphotyrosine antibody (Harwerth *et al.*, 1992). The results are shown in Figure 2b. Treatment of cells with EGF (lane 2), TGFα-ETA (lane 4) or scFv(FRP5)-TGFα-ETA (lane 5) led to a strong increase in the phosphotyrosine content of a protein corresponding in size with the 170 kDa EGFR, which was confirmed by reprobing the filter with an anti-EGFR serum (Figure 2b, bottom). PBS and the monovalent ErbB-2-specific scFv(FRP5)-ETA had no effect on the phosphotyrosine content of the receptor (lanes 1 and 3). The results show that in contrast to the ErbB-2-specific antibody toxin scFv(FRP5)-ETA, the bivalent scFv(FRP5)-TGFα-ETA and the monovalent TGFα-ETA bind to and activate EGFR.

### In vitro *cell killing activity of scFv(FRP5)-TGFα-ETA*

We tested the cell killing activity of the bispecific scFv(FRP5)-TGFα-ETA on human tumour cell lines expressing different levels of ErbB-2 and EGFR as shown in Table I. A431 human squamous cell carcinoma cells and the human

**Figure 2** (**a**) Binding of scFv(FRP5)-TGFα-ETA to recombinant extracellular domain of ErbB-2. Immobilised extracellular domain of ErbB-2 was incubated with scFv(FRP5)-ETA (□), scFv(FRP5)-TGFα-ETA (▒) or TGFα-ETA (■) at concentrations of 1 nM or 10 nM. The amount of specifically bound protein was measured after incubation with rabbit anti-ETA serum, followed by alkaline phosphatase-coupled goat anti-rabbit IgG and conversion of the phosphatase substrate *p*-nitrophenylphosphate as the absorbance at 405 nm. (**b**) ScFv(FRP5)-TGFα-ETA- and TGFα-ETA-induced tyrosine phosphorylation of EGFR in NE1 murine fibroblasts. The cells were grown in low serum for 16 h and then incubated with 1 μg $ml^{-1}$ scFv(FRP5)-ETA (lane 3), TGFα-ETA (lane 4) or scFv(FRP5)-TGFα-ETA (lane 5) for 10 min. Control cells were treated with PBS (lane 1) or 100 ng $ml^{-1}$ EGF (lane 2). Equal amounts of cell lysates were analysed by SDS–PAGE and immunoblotting with an anti-phosphotyrosine MAb, followed by incubation with an anti-mouse horseradish peroxidase-labelled antibody and chemiluminescent detection (top). The amount of EGFR loaded in each lane was analysed by reincubation of the filter with 12E EGFR specific antiserum (bottom). The position of the 170 kDa EGFR is indicated (EGFR). M, molecular weight standards.

**Table I** *In vitro* cell killing activity of ETA fusion proteins

| *Cell line* | *EGFR*[b] | *ErbB-2*[b] | *TGFα-ETA* | *scFv(FRP5)-ETA* | $IC_{50}$ *(nM)*[a] *scFv(FRP5)-TGFα-ETA* | $scFv_2$*(FRP5/225)-ETA* |
|---|---|---|---|---|---|---|
| A431 | +++++ | + | <0.02 | 0.52 | 0.04 | 0.02[c] |
| MDA-MB468 | +++++ | +/– | 0.02 | >15 | 0.42 | 3.04[c] |
| MDA-MB453 | +/– | ++++ | 0.53 | <0.01 | <0.01 | ND[d] |
| T47D | + | ++ | 2.23 | 0.13 | 0.82 | ND |
| SKBR3 | + | ++++ | 1.66 | 0.34[c] | 1.24 | 0.32[c] |

[a]$IC_{50}$ values were determined in an enzymatic cell viability assay as described in Materials and methods. The data are calculated from Figure 3. [b]Relative levels of receptor expression were determined by quantitative immunoblot analysis with ErbB-2- and EGFR-specific antibodies (not shown). [c]Schmidt *et al.* (1996). [d], not done.

**Figure 3** Inhibition of the growth of human tumour cell lines by recombinant single chain toxins. MDA-MB453, T47D and MDA-MB468 human breast carcinoma cells, and A431 human squamous cell carcinoma cells were incubated for 40 h with the indicated concentrations of scFv(FRP5)-TGFα-ETA (○), scFv(FRP5)-ETA (■), or with TGFα-ETA (▲). The relative number of viable cells was determined using an enzymatic assay described in Materials and methods and is indicated as the absorption at 590 nm. Each point represents the mean of a set of data determined in triplicate in three independent experiments.

breast carcinoma cell lines MDA-MB453, MDA-MB468 and T47D, were incubated for 40 h with various concentrations of the bispecific scFv(FRP5)-TGFα-ETA and the corresponding monospecific toxins, scFv(FRP5)-ETA and TGFα-ETA. The relative number of viable cells was determined with an enzymatic assay (Wels *et al.*, 1992). The results are shown in Figure 3 and the $IC_{50}$ values are summarised in Table I.

ScFv(FRP5)-TGFα-ETA was cytotoxic for the five cell lines tested. The monospecific scFv(FRP5)-ETA and the bispecific scFv(FRP5)-TGFα-ETA showed similar activity on MDA-MB453 cells overexpressing ErbB-2. Both toxins displayed a greater than 50-fold higher cell killing activity than the EGFR-specific TGFα-ETA, with $IC_{50}$ values of less than 0.01 nM *vs* 0.53 nM respectively. Similarly, T47D cells were more sensitive to the toxins containing the ErbB-2-specific scFv than they were to TGFα-ETA, with $IC_{50}$ values of 0.13, 0.82 and 2.23 nM for scFv(FRP5)-ETA, scFv(FRP5)-TGFα-ETA and TGFα-ETA respectively. On A431 cells expressing high levels of EGFR and low levels of ErbB-2, scFv(FRP5)-TGFα-ETA was 13 times more active than scFv(FRP5)-ETA, but less active than TGFα-ETA, with $IC_{50}$ values of 0.04, 0.52 and less than 0.02 nM respectively. This is in contrast to another bivalent ETA fusion protein, $scFv_2$(FRP5/225)-ETA, which contains two scFv domains specific for ErbB-2 and EGFR and was more active on A431 cells than either of the corresponding monospecific scFv-toxins (Schmidt *et al.*, 1996, and Table I). Treatment of A431 cells with EGF did not result in significant cell death at concentrations below 3 nM indicating that the high cell killing activity of the TGFα-containing fusion proteins observed at low concentrations was a result of their toxin domain (data not shown). As previously reported, EGFR-overexpressing MDA-MD468 cells were not sensitive to the ErbB-2-specific scFv(FRP5)-ETA at the concentrations tested (Wels *et al.*, 1995; Schmidt *et al.*, 1996). However, they were killed by TGFα-ETA and scFv(FRP5)-TGFα-ETA with $IC_{50}$ values of 0.02 and 0.42 nM respectively. ScFv(FRP5)-TGFα-ETA was approximately seven times more active than the previously described bispecific antibody toxin $scFv_2$(FRP5/225)-ETA on MDA-MB468 cells (Schmidt *et al.*, 1996). In contrast, scFv(FRP5)-TGFα-ETA was less active than $scFv_2$(FRP5/255)-ETA on A431 cells (Table I).

### *Competition of scFv(FRP5)-TGFα-ETA cytotoxicity*

Competition experiments were carried out in order to determine the contribution of the individual binding domains to the cytotoxic activity of the bispecific scFv(FRP5)-TGFα-ETA molecule. A431 cells were treated for 40 h with 100 ng $ml^{-1}$ scFv(FRP5)-TGFα-ETA in the absence or presence of a 70-fold molar excess of the EGFR-specific MAb 225, which has previously been shown to compete the binding of EGF and TGFα to EGFR (Kawamoto *et al.*, 1983), the parental ErbB-2-specific antibody FRP5 (Harwerth *et al.*, 1992), or a mixture of both. Cell viability was measured in comparison with PBS-treated cells. The results are shown in Figure 4. A total of 90.5% of the cells were killed by scFv(FRP5)-TGFα-ETA in the absence of competitor. In the presence of an excess of MAb 225, the cell killing activity was reduced resulting in 65.8% cell killing. The ErbB-2-specific MAb FRP5 alone had only little effect on scFv(FRP5)-TGFα-ETA activity (84.5%

**Figure 4** Inhibition of scFv(FRP5)-TGFα-ETA cell killing activity by competition with monoclonal antibodies. A431 human squamous cell carcinoma cells were incubated for 40 h with 100 ng ml$^{-1}$ scFV(FRP5)-TGFα-ETA without the addition of competitor or in the presence of a 70-fold molar excess of the ErbB-2-specific MAb FRP5, or EGFR-specific MAb 225, or a combination of both as indicated. The relativel number of viable cells was determined as described in Figure 3 and in Materials and methods. Each point was determined in triplicate. The standard deviation is represented by error bars.

cell killing), since A431 cells express approximately 100 times more EGF receptor than ErbB-2 and the EGF receptors remain accessible for the toxin. However, a mixture of both competing antibodies reduced the cell killing activity of scFv(FRP5)-TGFα-ETA even further than MAb 225 alone (36.5% cell killing). These results indicate that both binding domains contribute to the cell killing activity of scFv(FRP5)-TGFα-ETA, with the TGFα domain being more important for its activity on A431 cells, which express high levels of EGFR and low levels of ErbB-2. Similar competition experiments were carried out with the monospecific TGFα-ETA. As expected, only MAb 225, but not MAb FRP5, led to a reduction of TGFα-ETA cell killing activity (data not shown).

### *Activation of receptor tyrosine kinases upon scFv(FRP5)-TGFα-ETA binding*

Type I receptor tyrosine kinases undergo activation of their cytoplasmic kinase domain upon ligand binding and receptor dimerisation. Ligand-induced activation of EGFR in addition to the formation of EGFR homodimers also leads to the formation of EGFR/ErbB-2 heterodimers (Goldman *et al.*, 1990; Wada *et al.*, 1990), as well as EGFR/ErbB-3 heterodimers (Soltoff *et al.*, 1994). The bispecific antibody toxin $scFv_2$ (FRP5/225)-ETA, containing ErbB-2 and EGFR binding domains, activates both receptors in A431 cells which express high levels of EGFR and low levels of ErbB-2 (Schmidt *et al.*, 1996). In contrast, the corresponding monovalent antibody toxins specific for EGFR or ErbB-2 were unable to induce receptor activation.

We analysed the effect of TGFα-ETA and scFv(FRP5)-TGFα-ETA on the activation of EGFR in A431 cells. The cells were treated for 10 min at 37°C with scFv(FRP5)-TGFα-ETA, TGFα-ETA or scFv(FRP5)-ETA. Controls cells were treated with PBS or 100 ng ml$^{-1}$ EGF. Equal amounts of cell lysates were assayed for their phosphotyrosine content by SDS-PAGE and subsequent immunoblotting with a specific anti-phosphotyrosine antibody. The results are shown in Figure 5a. Treatment of cells with EGF (lane 2), TGFα-ETA (lane 4), or the bispecific scFv(FRP5)-TGFα-ETA (lane 5) led to a strong increase in the phosphotyrosine content of a protein corresponding in size with the 170 kDa EGFR, which was confirmed by reprobing the filter with an anti-EGFR serum (Figure 5a, bottom). PBS and the monovalent scFv(FRP5)-ETA protein had no effect on the phosphotyrosine content of the receptor (lanes 1 and 3). Similarly, after immunoprecipitation with an ErbB-2-specific antiserum and immunoblotting with anti-phosphotyrosine MAb, an increase in ErbB-2 phosphotyrosine content was detected in A431 cells treated with EGF, TGFα-ETA or bispecific scFv(FRP5)-TGFα-ETA, but not in cells treated with the monospecific scFv(FRP5)-ETA (data not shown).

The effects of several fusion toxins binding to EGFR, ErbB-2, or both, on the activation of ErbB-2 was analysed in SKBR3 cells. These cells express approximately $1 \times 10^6$ ErbB-2 and $9 \times 10^4$ EGFR molecules. The cells were treated for 10 min at 37°C with the bispecific antibody toxin $scFv_2$(FRP5/225)-ETA, scFv(FRP5)-TGFα-ETA, the monovalent antibody toxins, scFv(225)-ETA or scFv(FRP5)-ETA, or TGFα-ETA. Control cells were treated with PBS or 100 ng ml$^{-1}$ EGF. Phosphotyrosine content of cellular proteins was determined by immunoblot analysis (Figure 5b). A phosphotyrosine-containing protein corresponding in size with the 185 kDa ErbB-2 was already observed in unstimulated SKBR3 cells treated with PBS (lane 1). The identity of this protein was confirmed by reprobing the filter with an anti-ErbB-2 serum (Figure 5b, bottom). Treatment of cells with EGF (lane 2), TGFα-ETA (lane 6) or scFv(FRP5)-TGFα-ETA (lane 7) led to a further increase in ErbB-2 phosphotyrosine content. No significant effect on ErbB-2 activation was observed after treatment of cells with scFv(FRP5)-ETA (lane 3), scFv(225)-ETA (lane 4) or $scFv_2$(FRP5/225)-ETA (lane 5).

The effects of bispecific toxins on the activation of ErbB-2 was analysed further in MDA-MB453 cells. These cells express high levels of ErbB-2, ErbB-3 and ErbB-4, but very low levels of EGFR (Jeschke *et al.*, 1995). The cells were treated for 10 min at 37°C with $scFv_2$(FRP5/225)-ETA, scFv(FRP5)-TGFα-ETA, TGFα-ETA, or the previously described ErbB-3/ErbB-4-specific heregulin fusion protein HRGβ1-ETA (Jeschke *et al.*, 1995). Control cells were treated with PBS or 100 ng ml$^{-1}$ EGF. Phosphotyrosine content of cellular proteins was determined by immunoblot analysis (Figure 5c). Treatment of cells with HRGβ1-ETA (lane 6) led to a strong increase in the phosphotyrosine content of a protein corresponding in size with the 185 kDa ErbB-2, which was confirmed by reprobing the filter with an anti-ErbB-2 serum (Figure 5c, bottom). The bispecific toxins, $scFv_2$(FRP5/225)-ETA (lane 3) and scFv(FRP5)-TGFα-ETA (lane 4), weakly stimulated ErbB-2 phosphorylation, whereas PBS (lane 1), EGF (lane 2) and the monospecific TGFα-ETA had no effect on the phosphotyrosine content of the receptor in MDA-MB453 cells. The results show that the bispecific toxins, scFv(FRP5)-TGFα-ETA and $scFv_2$(FRP5/225)-ETA, and the monospecific growth factor toxins, TGFα-ETA and HRGβ1-ETA, but not the monospecific antibody toxin scFv(FRP5)-ETA, induce the activation of EGFR and/or ErbB-2. Thereby, the activity of the proteins is dependent on the cellular context and the expression level of ErbB receptor family members.

### *Inhibition of tumour cell growth* in vivo

The *in vivo* anti-tumour activity of scFv(FRP5)-TGFα-ETA and TGFα-ETA was tested on A431 xenografts in nude mice. A431 tumour tissue (25 μg) was implanted s.c. into three groups of five mice on day 0. Six days later, when the tumours had reached approximately 100 mm$^3$ in size, treatment was begun. The mice received twice daily intraperitoneal injections of 80 pmol of scFv(FRP5)-TGFα-ETA or TGFα-ETA for a total of 10 days. Control mice received PBS. The results are shown in Figure 6a. Treatment with both fusion toxins led to the inhibition of A431 tumour growth during treatment to a similar extent. By day 22, when the experiment was terminated, the size of the tumours in the scFv(FRP5)-TGFα-ETA and TGFα-ETA treated animals was, respectively, 21% and 22% of the tumour size in the control group. A transient weight loss of less than 10% was observed during the course of the TGFα-ETA treatment (Figure 6b). Weight loss of less than 5% was observed in the scFv(FRP5)-TGFα-ETA-treated animals. All animals recovered quickly after the end of the treatment.

**Figure 5** ScFv(FRP5)-TGFα-ETA and TGFα-ETA-induced tyrosine phosphorylation of EGFR and ErbB-2. (**a**) A431 cells were grown in low serum for 16 h and then incubated with 1 $\mu g\ ml^{-1}$ scFv(FRP5)-ETA (lane 3), TGFα-ETA (lane 4), or scFv(FRP5)-TGFα-ETA (lane 5) for 10 min. Control cells were treated with PBS (lane 1) or 100 $ng\ ml^{-1}$ EGF (lane 2). Equal amounts of cell lysates were analysed by SDS–PAGE and immunoblotting with an anti-phosphotyrosine MAb (top) or with 12E EGFR-specific antiserum (bottom) as described in the legend for Figure 2b. The position of the 170 kDa EGFR is indicated (EGFR). (**b**) SKBR3 human breast carcinoma cells were grown in low serum as described above and then treated with 1 $\mu g\ ml^{-1}$ scFv(FRP5)-ETA (lane 3), EGFR-specific antibody toxin scFv(225)-ETA (lane 4), the bispecific antibody toxin $scFv_2$(FRP5/225)-ETA (lane 5), TGFα-ETA (lane 6), or scFv(FRP5)-TGFα-ETA (lane 7) for 10 min. Control cells were treated with PBS (lane 1) or 100 $ng\ ml^{-1}$ EGF (lane 2). Equal amounts of cell lysates were analysed by SDS–PAGE and immunoblotting with an anti-phosphotyrosine MAb (top) as described in the legend for Figure 2b or with 21N ErbB-2-specific antiserum (bottom). The position of the 185 kDa ErbB-2 is indicated. (**c**) MDA-MB453 human breast carcinoma cells were grown in low serum as described above and then treated with 1 $\mu g\ ml^{-1}$ bispecific antibody toxin $scFv_2$(FRP5/225)-ETA (lane 3), EGFR-specific TGFα-ETA (lane 4), the bispecific scFv(FRP5)-TGFα-ETA (lane 5), or the ErbB-3/ErbB-4-specific heregulin fusion protein HRGβ1-ETA (lane 6) for 10 min. Control cells were treated with PBS (lane 1) or 100 $ng\ ml^{-1}$ EGF (lane 2). Equal amounts of cell lysates were analysed by SDS–PAGE and immunoblotting with an anti-phosphotyrosine MAb (top) as described in the legend for Figure 2b or with 21N ErbB-2-specific antiserum (bottom). The position of the 185 kDa ErbB-2 is indicated. M, molecular weight standards.

### Discussion

Overexpression of EGFR and ErbB-2 has been observed in a variety of human tumours making these receptors promising targets for directed tumour therapy (reviewed in Gullick, 1991; Hynes and Stern, 1994). Several recombinant *Pseudomonas* exotoxin A fusion proteins binding to EGFR or ErbB-2 have been described (Chaudhary *et al.*, 1987; Wels *et al.*, 1992, 1995; Batra *et al.*, 1992). Since many tumour cells express both ErbB-2 and EGFR, therapeutic reagents binding to both receptor proteins might offer advantages over monospecific molecules by inducing the formation of receptor heterodimers which in turn might lead to more rapid uptake of toxin–receptor complexes. We have recently characterised $scFv_2$(FRP5/225)-ETA, a bispecific antibody toxin, which contains, in a single polypeptide chain, ErbB-2- and EGFR-specific scFv domains linked to *Pseudomonas* exotoxin A (Schmidt *et al.*, 1996). This molecule was cytotoxic *in vitro* and *in vivo* for tumour cells expressing EGFR, ErbB-2 or both receptor proteins.

Here we describe the construction and functional characterisation of another bispecific recombinant toxin, scFv(FRP5)-TGFα-ETA binding to ErbB-2 and EGFR. The fusion protein consists of the antigen-binding domains

**Figure 6** Effect of recombinant single chain toxins on the *in vivo* growth of A431 tumour xenografts in nude mice. A431 tumour tissue (25 $\mu$g) were implanted subcutaneously into each mouse (5 mice per group). Six days later the mice received i.p. injections of 80 pmol TGF$\alpha$-ETA (▲) or scFv(FRP5)-TGF$\alpha$-ETA (○) twice daily for 10 days. The control group received PBS (■). (**a**) Tumour size and (**b**) body weight were measured at the indicated times and tumour volumes were calculated. The mean values for each group are shown.

of the ErbB-2-specific MAb FRP5 and the natural EGFR ligand TGF$\alpha$ linked to truncated *Pseudomonas* exotoxin A. We have shown that the scFv(FRP5)-TGF$\alpha$-ETA protein binds to EGFR and ErbB-2 and is cytotoxic for tumour cells expressing various levels of the target receptors. Despite the presence of two cell recognition domains this bispecific molecule is very similar in size to the parental ErbB-2-specific antibody toxin scFv(FRP5)-ETA (73 *vs* 67 kDa) and much smaller than the previously described bispecific antibody toxin scFv$_2$(FRP5/225)-ETA (107 kDa). This might allow better tumour penetration and faster blood clearance (Colcher *et al.*, 1990).

The different biological activities of ETA required for target cell recognition, translocation to the cytosol and enzymatic activity reside in separate protein domains which function independently (Hwang *et al.*, 1987). This allows the insertion of foreign protein sequences at the domain boundaries. Although insertion at certain positions can result in reduced cytotoxic activity, *Pseudomonas* exotoxin A has proved to be surprisingly flexible with regard to possible integration sites for small heterologous binding domains. ETA fusion proteins containing TGF$\alpha$, either N-terminal of the ETA translocation domain II (Siegall *et al.*, 1989) similar to TGF$\alpha$-ETA in our study, or at the C-terminus of the enzymatic domain III (Chaudhary *et al.*, 1987), have been derived and were biologically active. The activity of proteins with a heterologous binding domain inserted between domains II and III of ETA seems to be dependent upon the size and/or nature of the ligand. The 50 amino acid TGF$\alpha$ domain located between translocation and enzymatic domains in the bispecific scFv(FRP5)-TGF$\alpha$-ETA retains binding activity, whereas the 27 kDa ErbB-2-specific scFv(FRP5) domain inserted at the same position results in a fusion toxin with drastically reduced cytotoxic activity (Schmidt and Wels, unpublished data).

Internalisation of toxin-receptor complexes and subsequent intracellular processing of the ETA domain is a prerequisite for the cytotoxic activity of recombinant fusion toxins (Ogata *et al.*, 1992). TGF$\alpha$-containing growth factor toxins, but not the monospecific antibody toxins, scFv(FRP5)-ETA (anti-ErbB-2) and scFv(225)-ETA (anti-EGFR), activate ErbB-2 or EGFR upon binding, suggesting that the scFv-ETA proteins cannot induce receptor dimerisation and activation (Schmidt *et al.*, 1996). Cellular uptake of these antibody toxins therefore is dependent on the intrinsic turnover rate of the target receptors. In contrast, bispecific scFv(FRP5)-TGF$\alpha$-ETA induced tyrosine phosphorylation of both EGFR and ErbB-2. Activation of the receptors upon binding might also result in more rapid internalisation of the bispecific toxin. Since activation of ErbB-2 and EGFR to a similar degree was also observed after treatment of A431 and SKBR3 cells with monospecific TGF$\alpha$-ETA and EGF, it remains unclear to what extent EGFR/ErbB-2 heterodimerisation and activation in these cells is dependent on the additional ErbB-2-specific scFv domain in the bispecific molecule. In agreement with a previous report (Jeschke *et al.*, 1995) in MDA-MB453 cells expressing less than 5000 EGFR molecules, HRG$\beta$1-ETA, a fusion toxin containing the EGF-like domain of the ErbB-3/ErbB-4 ligand heregulin $\beta$1, but not TGF$\alpha$-ETA or EGF were able to induce tyrosine phosphorylation of ErbB-2. This suggests that in these cells transmodulation of ErbB-2 does not occur via interaction with EGFR but mostly via interaction with ErbB-3 and/or ErbB-4. Similar results have been observed in cells expressing either ErbB-2 and ErbB-3, or ErbB-2 and ErbB-4 in the complete absence of EGFR (Riese *et al.*, 1995). However, both bispecific toxins, scFv(FRP5)-TGF$\alpha$-ETA and scFv$_2$(FRP5/225)-ETA, were able to induce activation of ErbB-2 in MDA-MB453 cells. Thereby the much lower level of ErbB-2 tyrosine phosphorylation in comparison with that after treatment with HRG$\beta$1-ETA probably reflects the very limited numbers of EGFR which are present in MDA-MB453 cells and could be recruited for ErbB-2/EGFR heterodimers. Our data suggest that such artificial ligands, after removal of the toxic effector domain, might also be useful in studying signal transduction and growth-modulating activities of defined receptor heterodimers even when such dimers are not normally induced in a specific cell line by natural ligands of the ErbB receptor family.

Interestingly, scFv(FRP5)-TGF$\alpha$-ETA, despite the low abundancy of EGFR, displayed much higher cell killing activity on MDA-MB453 cells than on the other cell lines tested. Its activity equalled that of the ErbB-2-specific scFv(FRP5)-ETA, whereas on EGFR-overexpressing A431 and MDA-MB468 cells, bispecific scFv(FRP5)-TGF$\alpha$-ETA *in vitro* was less active than monospecific TGF$\alpha$-ETA. This suggests that reduced binding activity of the TGF$\alpha$ domain in the bispecific molecule rather than reduced scFv(FRP5) binding or a loss of activity of the ETA portion might be responsible. Similarly, a fusion protein containing TGF$\alpha$ and an anti-Tac scFv, both located at the N-terminus of ETA domains II, Ib and III, was less active on EGFR-overexpressing A431 cells than a monospecific TGF$\alpha$ fusion toxin (Batra *et al.*, 1990).

ScFv(FRP5)-TGFα-ETA displayed potent *in vivo* antitumour activity against established A431 xenografts in nude mice. In contrast to its activity on A431 cells *in vitro*, the bispecific molecule was as active *in vivo* as the monospecific TGFα-ETA. The observed anti-tumoral activity was specific for the fusion proteins since in a similar experiment, treatment of the animals with the truncated ETA portion alone lacking a cell binding domain had no effect on tumour growth (Schmidt *et al.*, unpublished results). Some normal tissues including hepatocytes express significant numbers of EGFR which could complicate the application of TGFα containing toxins *in vivo* (Real *et al.*, 1986). Systemic treatment of mice with high doses of TGFα-PE40, a fusion toxin very similar to the TGFα-ETA used in this study, resulted in fatal liver damage, thus limiting the amount of toxin which could be applied safely (Pai *et al.*, 1991). In order to avoid systemic toxicity in a recent clinical study of TGFα-PE40 (TP40) in superficial bladder cancer, the molecule was applied directly into the bladder by transurethral instillation (Goldberg *et al.*, 1995). The treatment was well tolerated by the patients indicating that local treatment, where applicable, could be a way to circumvent systemic toxicity. In our study there were no fatal effects in mice after i.p. injection of TGFα-ETA at the applied dose but we observed weight loss which was transient and subsided quickly after treatment was terminated. Weight loss was also observed in animals treated with the bispecific scFv(FRP5)-TGFα-ETA, but it was less pronounced suggesting that normal tissues expressing EGFR might tolerate the bispecific molecule better. This could be due to reduced binding activity of the intramolecular TGFα domain and/or improved tumour targeting owing to the additional tumour-specific scFv domain.

Our results show that a scFv antibody domain and the TGFα growth factor domain inserted at different locations in *Pseudomonas* exotoxin A result in a bispecific toxin which binds to both ErbB-2 and EGFR tyrosine kinases. The fusion protein displays *in vitro* and *in vivo* cell killing activity on human tumour cells expressing both target antigens on their surface. The coexpression of ErbB-2 and EGFR observed in many human tumours and their synergistic interaction in the transformation of cells provides a rationale for the further development of such bispecific reagents for clinical applications.

**Acknowledgements**
The authors thank Drs N Hynes and B Groner for helpful discussions, Dr M Jeschke for providing purified recombinant ErbB-2 extracellular domain, and Mr M Müller for organisation of the animal experiments. This work was supported in part by a grant from the Deutsche Forschungsgemeinschaft (SFB 364-CI).

### References

BATRA JK, CHAUDHARY VK, FITZGERALD D AND PASTAN I. (1990). TGFα-anti.Tac(Fv)-PE40: a bifunctional toxin cytotoxic for cells with EGF or IL2 receptors. *Biochem. Biophys. Res. Commun.*, **171**, 1-6.

BATRA JK, KASPRZYK PG, BIRD RE, PASTAN I AND KING CR. (1992). Recombinant anti-erbB-2 immunotoxins containing *Pseudomonas* exotoxin. *Proc. Natl Acad. Sci. USA*, **89**, 5867-5871.

BEERLI RR, WELS W AND HYNES NE. (1994). Autocrine inhibition of the epidermal growth factor by intracellular expression of a single-chain antibody. *Biochem. Biophys. Res. Commun.*, **204**, 666-672.

CHAUDHARY VK, FITZGERALD DJ, ADHYA S AND PASTAN I. (1987). Activity of a recombinant fusion protein between transforming growth factor type α and Pseudomonas toxin. *Proc. Natl Acad. Sci. USA*, **84**, 4538-4542.

CHOMCZYNSKI P AND SACCHI N. (1987). Single-step method of RNA isolation by acid guanidium thiocyanate-phenol-chloroform extraction. *Anal. Biochem.*, **162**, 156-159.

COLCHER D, BIRD R, ROSELLI M, HARDMAN KD, JOHNSON S, POPE S, DODD SW, PANTOLIANO MW, MILENIC DE AND SCHLOM J. (1990). *In vivo* tumor targeting of a recombinant single-chain antigen-binding protein. *J. Natl Cancer Inst.*, **82**, 1191-1197.

GOLDBERG MR, HEIMBROOK DC, RUSSO P, SAROSDY MF, GREENBERG RE, GIANTONIO BJ, LINEHAN WM, WALTHER M, FISHER HAG, MESSING E, CRAWFORD ED, OLIFF AI AND PASTAN IH. (1995). Phase I clinical study of the recombinant oncotoxin TP40 in superficial bladder cancer. *Clin. Cancer Res.*, **1**, 57-61.

GOLDMAN R, BEN LEVI R, PELES E AND YARDEN Y. (1990). Heterodimerization of the erbB-1 and erbB-2 receptors in human breast carcinoma cells: a mechanism for receptor transregulation. *Biochemistry*, **29**, 11024-11028.

GULLICK WJ. (1991). Prevalence of abberant expression of the epidermal growth factor receptor in human cancers. *Br. Med. Bull.*, **47**, 87-98.

HARWERTH IM, WELS W, MARTE BM AND HYNES NE. (1992). Monoclonal antibodies against the extracellular domain of the erbB-2 receptor function as a partial ligand agonists. *J. Biol. Chem.*, **267**, 15160-15167.

HWANG J, FITZGERALD DJ, ADHYA S AND PASTAN I. (1987). Functional domains of *Pseudomonas* exotoxin identified by deletion analysis of the gene expressed in *E coli*. *Cell*, **48**, 129-136.

HYNES NE AND STERN DF. (1994). The biology of erbB-2/neu/HER-2 and its role in cancer. *Biochim. Biophys. Acta*, **1198**, 165-184.

JESCHKE M, WELS W, DENGLER W, IMBER R, STÖCKLIN E AND GRONER B. (1995). Targeted inhibition of tumor-cell growth by recombinant heregulin-toxin fusion proteins. *Int. J. Cancer*, **60**, 730-739.

KAWAMOTO T, SATO JD, LE A, POLIKOFF J, SATO GH AND MENDELSOHN J. (1983). Growth stimulation of A431 cells by epidermal growth factor: identification of high-affinity receptors for epidermal growth factor by an anti-receptor monoclonal antibody. *Proc. Natl Acad. Sci. USA*, **80**, 1337-1341.

KING CR, BORELLO I, BELLOT F, COMOGLIO P AND SCHLESSINGER J. (1988). EGF binding to its receptor triggers a rapid tyrosine phosphorylation of the *erb*B-2 protein in the mammary tumour cell line SK-BR-3. *EMBO J.*, **7**, 1647-1651.

KOKAI Y, MYERS JN, WADA T, BROWN VI, LEVEA CM, DAVIS JG, DOBASHI K AND GREENE MI. (1989). Synergistic interaction of $p185^{c-neu}$ and the EGF receptor leads to transformation of rodent fibroblasts. *Cell*, **58**, 287-292.

OGATA M, FRYLING CM, PASTAN I AND FITZGERALD DJ. (1992). Cell-mediated cleavage of *Pseudomonas* exotoxin between Arg279 and Gly280 generates the enzymatically active fragment which translocates to the cytosol. *J. Biol. Chem.*, **267**, 25396-25401.

PAI LH, GALLO MG, FITZGERALD DJ AND PASTAN I. (1991). Antitumor activity of a transforming growth factor α-*Pseudomonas* exotoxin fusion protein (TGF-α-PE40). *Cancer Res.*, **51**, 2808-2812.

PELES E AND YARDEN Y. (1993). Neu and its ligands: from an oncogene to neural factors. *Bioessays*, **15**, 815-824.

PLOWMAN GD, GREEN JM, CULOUSCOU JM, CARLTON GW, ROTHWELL VM AND BUCKLEY S. (1993). Heregulin induces tyrosine phosphorylation of $HER4/p180^{erbB4}$. *Nature*, **366**, 473-475.

REAL F, RETTIG W, CHESA P, MELAMED MR, OLD LJ AND MENDELSOHN J. (1986). Expression of epidermal growth factor receptor in human cultured cells and tissues: relationship to cell lineage and stage of differentiation. *Cancer Res.*, **46**, 4726-4731.

RIESE DJ II, VAN RAAIJ TM, PLOWMAN GD, ANDREWS GC AND STERN DF. (1995). The cellular response to neuregulins is governed by complex interactions of the erbB receptor family. *Mol. Cell. Biol.*, **15**, 5770-5776.

SCHMIDT M, HYNES NE, GRONER B AND WELS W. (1996). A bivalent single chain antibody-toxin specific for ErbB-2 and the EGF receptor. *Int. J. Cancer*, **65**, 538-546.

SIEGALL CB, XU YH, CHAUDHARY VK, ADHYA S, FITZGERALD D AND PASTAN I. (1989). Cytotoxic activities of a fusion protein comprised of TGFα and *Pseudomonas* exotoxin. *FASEB J.*, **3**, 2647-2652.

SLIWKOWSKI MX, SCHAEFER G, AKITA RW, LOFGREN JA, FITZPATRICK VD, NUIJENS A, FENDLY BM, CERIONE RA, VANDLEN RL AND CARRAWAY KL III. (1994). Coexpression of *erbB2* and *erbB3* proteins reconstitutes a high affinity receptor for heregulin. *J. Biol. Chem.*, **269,** 14661-14665.

SOLTOFF SP, CARRAWAY KL III, PRIGENT SA, GULLICK WG AND CANTLEY LC. (1994). ErbB3 is involved in activation of phosphatidylinositol 3-kinase by epidermal growth factor. *Mol. Cell. Biol.*, **14,** 3550-3558.

WADA T, QIAN X AND GREEN MI. (1990). Intermolecular association of the $p185^{neu}$ protein and the EGF receptor modulates EGF receptor function. *Cell*, **61,** 1339-1347.

WELS W, HARWERTH IM, MUELLER M, GRONER B AND HYNES NE. (1992). Selective inhibition of tumor cell growth by a recombinant single-chain antibody-toxin specific for the erbB-2 receptor. *Cancer Res.*, **52,** 6310-6317.